# Progress in physical activity research, policy, and surveillance in Canada: The global observatory for physical activity – GoPA!

**DOI:** 10.1186/s12889-024-20322-1

**Published:** 2024-10-17

**Authors:** Ashley Cathro, John C Spence, Christine Cameron, Andrea Ramirez Varela, Diana Morales, Eduardo Ribes Kohn, Michael Pratt, Pedro C Hallal

**Affiliations:** 1 Department of Health and Kinesiology, University of Illinois Urbana-Champaign, Freer Hall - 906 S Goodwin Ave, Urbana, IL 61801 USA; 2 Faculty of Kinesiology, Sport, and Recreation, University of Alberta, Edmonton, Canada; 3 Canadian Fitness and Lifestyle Research Institute, Ottawa, Canada; 4 Department of Epidemiology, Center for Pediatric Population Health, Department of Pediatrics at McGovern Medical School, UTHealth Houston School of Public Health, University of Texas, Houston, US; 5 School of Physical Education, Federal University of Pelotas, Pelotas, RS Brazil; 6 Herbert Wertheim School of Public Health & Human Longevity Science, University of California San Diego, San Diego, US

**Keywords:** Public health, Exercise, Review

## Abstract

**Background:**

The purpose of this paper is to examine the evolution of physical activity research and the comprehensiveness of national physical activity policies and surveillance systems in Canada.

**Methods:**

A systematic review was conducted by the Global Observatory for Physical Activity (GoPA! ) on physical activity and health publications between 1950 and 2019. Findings from Canada were extracted and included in the present analysis. The number of articles published, female researcher involvement in authorship, author institution affiliations, and publication themes were examined. Policies were evaluated by determining if there was a standalone physical activity plan and if national guidelines existed. Surveillance systems were assessed for periodicity, instruments used, and age inclusivity.

**Results:**

Out of 23,000 + publications analyzed worldwide; 1,962 included data collected in Canada. Physical activity research in Canada increased considerably from the 2000s to 2010s (543 articles vs. 1,288 articles), but an apparent stabilization has been observed more recently. Most physical activity publications in Canada focused on surveillance (37%), with fewer articles on policy (8%) and interventions (7%). The proportion of female first authors increased from 38% in the 1980s to 60% in the last decade. However, females remain the minority for senior authors. With respect to policy, “A Common Vision” is Canada’s national plan, which has a singular policy focus on physical activity. National surveillance data is collected regularly with both the Canadian Health Measures Survey (CHMS) and the Canadian Community Health Survey. In addition to self-report, the CHMS also collects accelerometer data from participants.

**Conclusion:**

Through collaborative and coordinated action, Canada remains well equipped to tackle physical inactivity. Continued efforts are needed to enhance sustained awareness of existing physical activity promotion resources to increase physical activity.

## Introduction

Over the past 20 years, there has been a growing recognition that physical activity plays an important role in reducing the risk for non-communicable diseases such as diabetes, cardiovascular disease, and certain forms of cancer [1]. While it is well documented that physical activity can improve quality of life [2], physical activity rates in Canada remain suboptimal. When considering the 2020 physical activity targets as recommended by the Canadian 24-Hour Movement Guidelines which state adults should accumulate 150 min of moderate-to-vigorous intensity physical activity (MVPA) weekly and children and youth should achieve 60 min daily, accelerometer data indicates that only 49.2% of adults and 43.9% of children and youth met these recommended guidelines respectively [3]. In this regard, physical inactivity remains a challenge in Canada, as well as a significant global health concern, being responsible for > 5 million deaths per year globally [4]. Although physical inactivity may be perceived as an individual’s problem, it can have significant implications at the societal level as well. As a result, the 2022 direct and indirect health care costs of physical inactivity in Canada combined for a total of at least $3.9 billion (CAD) [5]. However, if as little as 10% of Canadians can improve upon suboptimal levels of physical activity and sedentary behavior, it could yield the Canadian health care system $2.6 billion (CAD) in cost-savings by 2040 [6]. 

Located in North America, Canada is comprised of ten provinces and three territories and is ranked 38th by population globally [7]. As of July 1, 2023, the total population estimate of Canada was 40,097,761 with the country most recently experiencing a 2.9% population growth rate from 2022 to 2023 [8]. This marked the highest growth rate for 12 months since 1957. While life expectancy in Canada has steadily increased over the past decade, the COVID-19 pandemic halted this trend by contributing to the largest single-year decline in life expectancy (i.e., a decrease of 0.6 years in 2020[9]). When considering the self-reported overall health of Canadians aged 12 and older, more than half reported their health to be very good or excellent [10]. However, almost half of Canadians (45.1%) also reported having one of the following common chronic diseases: diabetes, high blood pressure, heart disease, cancer, arthritis, stroke, mood disorders, or anxiety [11]. Furthermore, with the increasing prevalence and number of chronic conditions with age, coupled with the ongoing demographic shift towards an aging population, healthcare expenditures are expected to increase substantially [12]. This places an even greater need for the implementation of effective physical activity programs, interventions, and policies.

Canada has long demonstrated an interest in supporting physical activity policy, surveillance, and research. Since the late 1800s, physical activity has been recognized in the national policy agenda [13]. Over that period, the relevance or interests in physical activity was due to concerns about the physical fitness of young men for service in the armed forces, addressing social issues associated with economic recessions, fulfilling expectations around elite sport competitions (e.g., the Olympics), and, more recently, recognizing the public health burden of chronic diseases [14]. The 1970s through the 1980s was a significant period for physical activity in the country. For instance, along with important policy documents, ParticipACTION was established in 1971 to serve as a social marketing voice for physical activity [15] and the Canadian Fitness and Lifestyle Research Institute (CFLRI) was established in 1980 to monitor prevalence and correlates of physical activity, sport, and recreation of Canadians [16]. Both organizations continue to operate and, along with many others, contribute to a sector-wide approach to fostering physical activity opportunities for all. More recently, other important initiatives include the application of a systematic process for developing physical activity guidelines [17] and the employ of a report card to evaluate and document progress on efforts to facilitate physical activity among Canadians [18]. 

In the sciences field, research has shown that research groups that are gender diverse have the capacity to not only increase research productivity but also novelty [19]. Despite this, women are often underrepresented especially in leadership positions, and this can negatively impact their career progression [20]. Analyzing the proportion of female first and senior authors over time helps provide insight regarding progress made into achieving gender equity within the research community. By further understanding these trends in authorship, polices and initiatives can be implemented to support equity and diminish gender disparities.

To help reduce the global burden of physical inactivity, the Global Observatory for Physical Activity (GoPA! ) was initiated in 2012 and is dedicated to monitoring physical activity surveillance, research, and policy worldwide. In 2015, GoPA! created the first edition of country cards for 139 countries, which included physical activity indicator data on surveillance, research, and policy [21, 22]. The country cards serve as an invaluable tool to advocate for change and with concentrated efforts that focus on implementing evidence-based strategies to increase physical activity. A second set of cards was launched in 2020 and included 164 countries [23]. 

The purpose of this paper is to evaluate progress in physical activity research, policy, and surveillance in Canada.

## Methods

In this analysis, we used information extracted from a systematic review of all physical activity and health publications available from the PubMed, SCOPUS and ISI Web of Knowledge databases to assess the status of physical activity research [24]. For the review, the dates of publication were restricted to 1950–2019, and the search terms used included physical activity in the title or abstract and country name in English. Papers that focused on physical activity using sports or exercise key words were also included. Further details about the methods used for the systematic review can be found elsewhere [24]. Of the > 23,000 articles identified by GoPA! that met the inclusion criteria, 1,962 articles were attributed to Canada. This attribution was based on the inclusion of data collected either exclusively in Canada or as part of a multinational study incorporating data collected in Canada.

In terms of physical activity research in Canada, we primarily focused on examining: (a) the number of physical activity articles published by year, regardless of study type; (b) the proportion of articles including a female author; (c) the proportion of articles with a female first author; (d) the proportion of articles with a female senior author; (e) the institution of affiliation of authors; and (f) the theme of publication. Each article was categorized into one of five themes: surveillance, correlates and determinants, health consequences, interventions, or policy [24]. An article was classified as surveillance if it discussed physical activity levels, physical activity trends, or the measurement of physical activity. Correlates and determinants consisted of studies that examined the association between physical activity and various factors at the individual, interpersonal, environmental, and policy levels. Health consequences included articles related to the impact of physical activity on health outcomes. Studies that assessed the impact and effectiveness of strategies to promote physical activity were placed in the interventions category. Studies that focused on physical activity policy, physical activity guidelines, or the assessment of physical activity policy indicators were placed in the policy category. From the articles extracted in the systematic review, a database with bibliometric variables was created using Google Forms and authors were classified as first authors or senior authors based on the authorship order in the publication [25]. The sex of the author was determined by searching the author’s name using social media, university websites, and government websites [25]. Data obtained from both the 2015 and 2020 Canada country cards produced by GoPA! were also used to assess how physical activity research, surveillance, and policy have changed over time.

For policy, we used information available in the GoPA! Canada country card and evaluated the existence of a physical activity strategy or plan and whether it was a standalone physical activity plan or embedded within a larger noncommunicable disease (NCD) or obesity plan. We also examined whether national guidelines for physical activity existed and if they covered various age groups and special populations.

Regarding physical activity surveillance which involves the ongoing, systematic collection and analysis of physical activity related data [26], we analyzed the existing national level surveillance systems, their periodicity, age ranges included, and types of measurement of physical activity used. Due to Canada’s recent focus on 24-hour movement guidelines [27–29] which recognize the interrelationship between physical activity, sleep, and sedentary behavior, we analyzed when these concepts were introduced into both policy and surveillance measures.

## Results

### Research

**Fig. 1:**
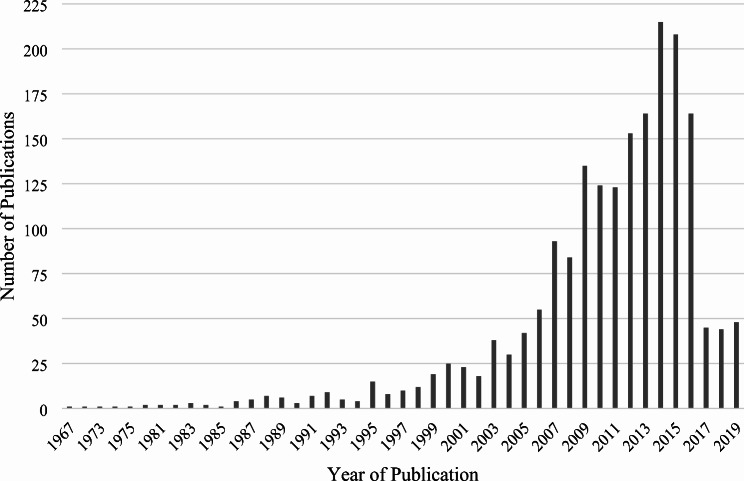
Number of physical activity publications in Canada by year

**Fig. 2:**
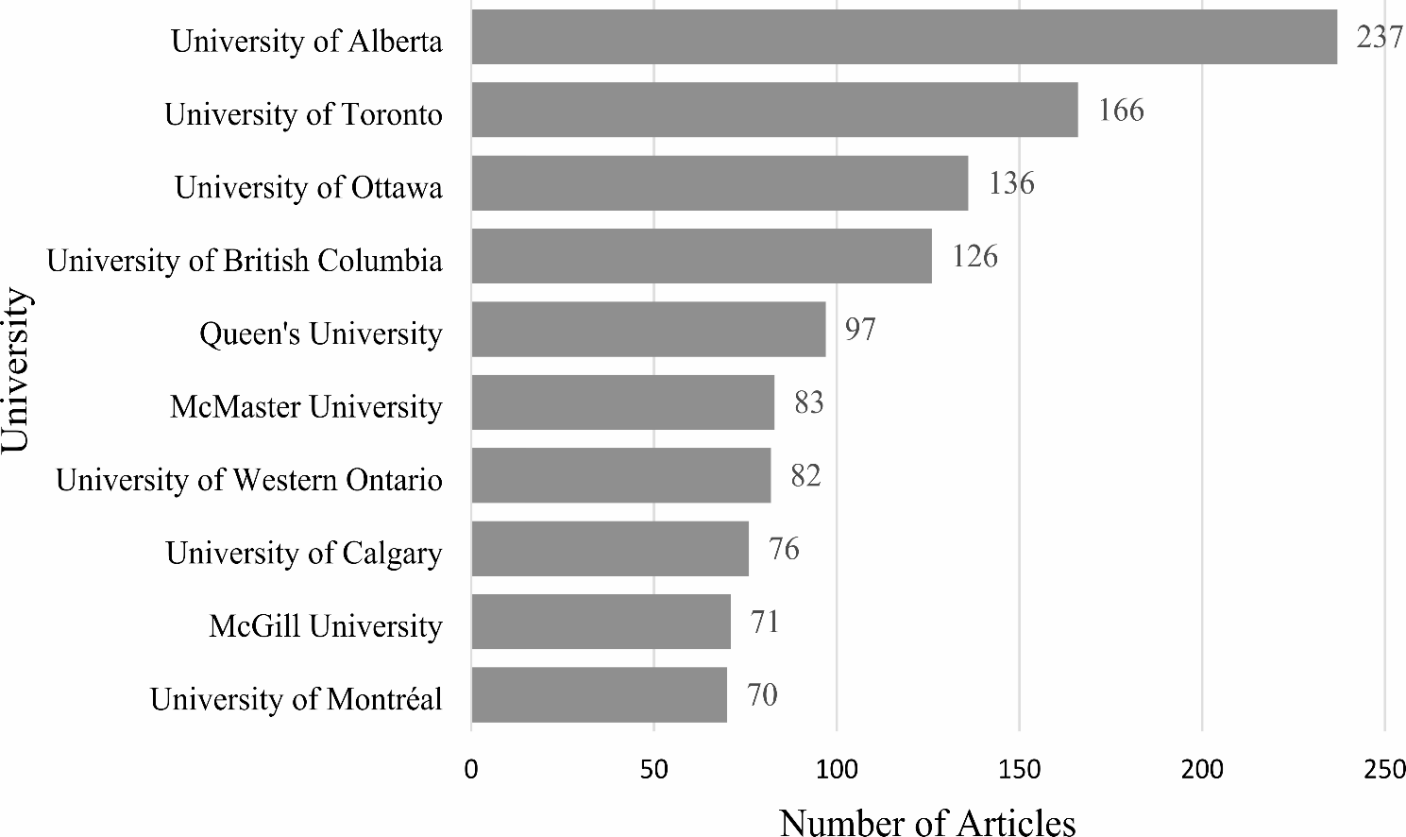
Number of articles published by the 10 Canadian institutions with the most physical activity and health publications

When considering physical activity publications in Canada from 1950 to 2019, 1,962 articles met the inclusion criteria as established by GoPA!. From 1950 to 2019, contributions to physical activity research have continued to grow. Although the dates of the articles reviewed are from 1950 to 2019, the first article was not published until 1967. Of the 1,962 articles published in Canada, 27.7% were published between 2000 and 2009, and 65.7% from 2010 to 2019 (Table 1). However, when articles published in the 2010s are broken down to publications annually, from 2014 onward, there appears to be a decline in publications (Fig. 1). Given Canada’s significant contributions to physical activity research, 45 Canadian universities are represented within the 1,962 published physical activity articles in Canada. As highlighted in Fig. 2, the top Canadian physical activity research-producing institution is the University of Alberta (237), followed by the University of Toronto (166) and the University of Ottawa (136). When considering the number of physical activity publications from the top 10 most highly producing institutions, over half (*n* = 1,144; 58.3%) come from these institutions.

**Table 1:** Number of Canadian physical activity and health publications by decade (1960–2019)

Decade	Number of publications	% of all publications
1960–1970 s	5	0.3%
1980s	34	1.7%
1990s	92	4.7%
2000s	543	27.7%
2010s	1288	65.6%

**Fig. 3:**
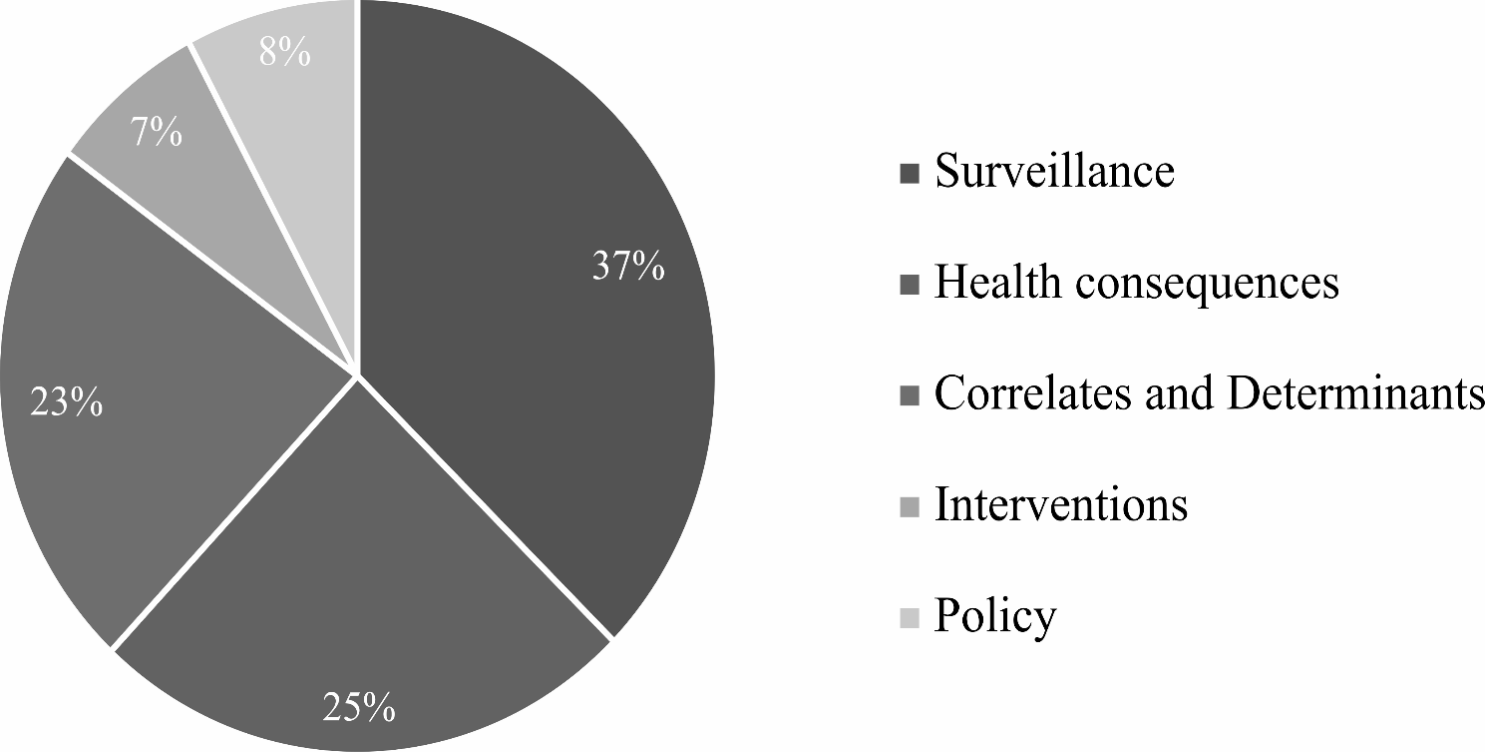
Physical activity publications in Canada categorized based on the proportion of each study theme

Physical activity publications in Canada can also be categorized by theme of study, as illustrated in Fig. 3. The greatest proportion of publications are on surveillance (physical activity prevalence, measurement, and time trends). Of the total publications in Canada, 37% fit within this category. This is followed by correlates and determinants of physical activity and health consequences of physical activity, which together account for nearly 50% of the publications. Lastly, both physical activity interventions and physical activity policy studies are the least frequently conducted themes in Canada, despite the almost 50 articles identified on Canada’s signature ParticipACTION program.

Female authorship has continued to become more prevalent in recent years, as 89.4% of published articles between 2010 and 2019 contained at least one female author (Table 2). An increase has occurred in the first author being a female by decade as illustrated in Table 1. For example, in the 1990s, 92 physical activity articles were published, and only 37% included a first author who was female. In the 2010s, however, 59.9% of the 1,288 papers published had a female first author.

**Table 2:** Female participation in authorship of physical activity and health articles from Canada. N (%)

			Decade			
	1960–1970 s	1980s	1990s	2000s	2010s	Total
**At Least 1 Female**	0 (0.0)	18 (52.9)	53 (57.6)	422 (77.7)	1151 (89.4)	1,644 (83.8)
**Female First Author**	0 (0.0)	13 (38.2)	34 (37.0)	261 (48.1)	771 (59.9)	1,079 (55.0)
**Female Senior Author**	0 (0.0)	9 (26.5)	29 (31.5)	211 (38.9)	523 (40.6)	772 (39.4)

Conversely, for senior authors, there were more males, with 60.7% of the published articles having a male senior author. Similar to first author trends, we are continuing to see a rise in female senior authors. However, this increase is occurring at a much lower rate. From 2000 to 2009, 38.9% of the published articles involved female senior authors (Table 2), whereas from 2010 to 2019, this percentage only increased to 40.6%.

### Policy

Canada is in a fortunate position in which it has multiple policies related to sport, physical activity, and recreation. In 2018, a first-of-its-kind national strategy document known as A Common Vision for Increasing Physical Activity and Reducing Sedentary Living in Canada: Let’s Get Moving [30] was published to encourage Canadians to move more and sit less. This standalone physical activity plan is Canada’s first to focus solely on physical activity and its dynamic relationship with sport, recreation, and health [30]. Moreover, it aims to build upon and amplify the efforts of other Canadian strategies including the Canadian Sport Policy 2012 [31], Active Canada 20/20,[32] the Framework for Recreation in Canada 2015 [33], and the National Active Transportation Strategy [34]. 

The Common Vision recognizes that many interrelated factors influence physical activity, and to address this complex issue, collaboration across various sectors and the orders of government are needed to increase physical activity at the national level [30]. The core of the strategy is based on six areas of focus, including cultural norms, spaces and places, public engagement, partnerships, leadership and learning, and progress. Each focus area is associated with strategic imperatives that act as a call to action for those with a stake in physical activity in Canada.

In terms of physical activity guidelines, Canada’s guidelines are comprehensive in nature. In 2016, The Canadian 24-Hour Movement Guidelines for Children and Youth (5–17 years) [28] were launched. These guidelines are the world’s first evidence-based guidelines that recognize the interrelationship between physical activity, sleep, and sedentary behavior over a 24-hour period [35]. In 2017, similar guidelines were published for the Early Years (0–4 years) [27], and in 2020 for adults (18–64 years and 65 years or older) [29]. These guidelines include three core recommendations to keep in mind, as set forward by The Canadian Society for Exercise Physiology (CSEP). They consist of moving more, reducing sedentary time, and sleeping well. Regardless of age group, the guidelines are relevant to apparently healthy individuals regardless of gender, race, ethnicity, or socio-economic status [27–29]. Aside from the current recommendations that exist for various age groups across the lifespan, guidelines also exist for special populations, such as adults with multiple sclerosis (MS) [36], adults with spinal cord injuries [37], and guidelines for physical activity through pregnancy [38]. While these other guidelines specifically focus on evidence-based physical activity recommendations, they do not include information on sleep and sedentary behavior. The specific guidelines for each population can be found in Table 3.

**Table 3:** 24-Hour Movement guidelines for different populations in Canada [27–29, 36–38].

	Physical Activity	Sleep	Sedentary Behavior
Early Years Infants (< 1)	- variety of ways, multiple times daily through interactive floor-based play - >30 min tummy time for those not mobile yet	− 14 to 17 h (aged 0–3 months) − 12 to 16 h (aged 4–11 months)	- not restrained in chair for > 1 h - no screen time
*Toddlers* *(1–2)*	- at least 180 min in a variety of activities daily at any intensity, include energetic play	− 11 to 14 h	- not restrained for > 1 h at a time - <2 years old no screen time, no more than 1 h for those aged 2
*Preschoolers (3–4)*	- at least 180 min with at least 60 min being energetic play per day	− 10 to 13 h	- not restrained for > 1 h at a time or sitting for extended periods - no more than 1 h screen time
**Children and Youth (5–17)**	- at least 60 min of MVPA per day involving aerobic activities - vigorous physical activity, and muscle and bone strengthening activities at least 3 days/week - several hours of structured and unstructured light physical activities	− 9 to 11 h uninterrupted for those aged 5–13 -8 to 10 h for those aged 14–17	- maximum of 2 h of recreational screen time/day - limit sitting for extended time periods
**Adults** **(18–64)**	- at least 150 min of aerobic MVPA per week - muscle strengthening activities at least 2 times/week - several hours of light physical activity, including standing	− 7 to 9 h	- limit sedentary time to 8 h or less including: - no more than 3 h of recreation screen time - breaking up long sitting periods
**Adults (65 +)**	- at least 150 min of aerobic MVPA per week - muscle strengthening activities at least 2 times/week - physical activities that challenge balance - several hours of light physical activity, including standing	− 7 to 8 h	- limit sedentary time to 8 h or less including: - no more than 3 h of recreation screen time - breaking up long sitting periods
**Pregnancy**	- at least 150 min of moderate intensity physical activity per week, accumulated over a minimum of 3 days - consider incorporating aerobic, resistance, and pelvic floor training		
**Multiple Sclerosis (18–64)**	− 30 min of moderate intensity aerobic exercise 2 times/week - strength training for major muscle groups 2 times/week		
**Adults with Spinal Cord Injuries**	- starting level: 20 min of aerobic MVPA 2 times/week and 3 sets of 10 reps strength training for major muscle groups 2 times/week - advanced level: 30 min of aerobic MVPA 3 times/week and 3 sets of 10 reps strength training for major muscle groups 2 times/week		

### Surveillance

Multiple physical activity surveillance systems exist with clear periodicity that has led GoPA! to classify Canada’s capacity for physical activity promotion in surveillance as high. The two primary sources of data listed on the GoPA! Canada country card include the Canadian Community Health Survey (CCHS) and the Canadian Health Measures Survey (CHMS). The CCHS is a voluntary, cross-sectional survey that first began in 2001 and gathers information related to health status, health care utilization and health determinants [39]. The survey is offered in both of Canada’s official languages, English and French, and provides an annual microdata file in addition to estimates at the health region level by combining files every two years. Moreover, in 2022 the survey transitioned from being face-to-face or by telephone to an online electronic questionnaire with its target population being individuals 18 years or older who live in the 10 provinces and the three territories. However, individuals who lived on reserves or in Aboriginal settlements, are full-time members of the Canadian Forces, institutionalized, or lived in Quebec health regions of Région du Nunavik and Région des Terres-Cries-de-la-Baie-James were excluded from participating in the survey.

Similar to the CCHS, Statistics Canada, in partnership with Health Canada and the Public Health Agency conducts the CHMS. The main goal of this surveillance system is to enhance the prevention, diagnosis, and treatment of diseases in Canada and ultimately improve the overall health of Canadians [40]. The CHMS has a target population of Canadians aged 1 to 79 with data collection occurring in two parts every two years. The population excluded from this survey is the same as the CCHS in addition to people living in the three territories. Regarding the methodology employed for conducting the CHMS, participants first complete a personal interview at their residence that includes questions pertaining to, but not limited to, lifestyle, physical activity, nutrition, and medical background. Following the interview, participants then schedule an appointment at a Mobile Examination Center (MEC), where a certified health specialist will take anthropometric measurements, assess cardiovascular health and fitness, and collect specimens among other measurements. By providing direct physical measures in addition to lifestyle characteristics, researchers can establish if correlations exist between certain risk factors and health outcomes. Following the visit at the MEC, respondents are provided with a waterproof accelerometer that is to be worn for seven days to track physical activity, stationary time, and sleep.

The CCHS and CHMS are used in GoPA! country cards due to comparability with other countries. However, physical activity surveillance in Canada operates through other institutions and mechanisms as well. For instance, the CFLRI is the only organization that dedicates itself to the surveillance and monitoring of physical activity, sport, and recreation in the country. This singular focus provides a comprehensive set of data to inform evidence-based policy and practice by examining correlates of physical activity, including policy, supportive environments, programming, capacity, and governance in major settings where activity takes place, and among the diverse populations across Canada [16]. 

## Discussion

To the best of our knowledge, this is the first review of the literature with the purpose of jointly analyzing physical activity research, policy, and surveillance progress among people of all ages in Canada. The examination of physical activity publications from 1950 to 2019 revealed a substantial growth in contributions over time. Although Canada’s population is only equivalent to 0.49% of the total world population [7], Canadian researchers have published the second most physical activity papers globally (8.3% of all publications) [41] which speaks to the country’s dedication to reducing the burden of physical inactivity. These findings parallel successful programs taking place in Canada (e.g., ParticipACTION, Sport for Life) and suggests that continued research efforts that focus on interventions aimed at promoting physical activity may be well received by the population. For instance, 55% of Canadian adults consider physical inactivity to be a serious public health concern [42]. In terms of surveillance, Canada relies on multiple means to assess physical activity, including self-reports and direct measures. Moreover, by including settings (e.g., schools, communities, workplaces) across the lifespan, along with clear periodicity in collecting data, it becomes easier to understand physical activity trends and disparities that may exist. Likewise, Canada recognizes the importance of having distinct physical activity, sedentary behavior, and sleep guidelines for different age groups as they help contribute to overall health and well-being.

The distribution of physical activity publications by the five major themes in Canada is similar to that of global trends [24]. Both in Canada (37.4%) and globally (32.5%),^24^ the largest proportion of publications are on physical activity prevalence, measurement, and trends. This is then followed by health consequences. With physical activity research being a relatively new field in comparison to other areas and disciplines, this explains why more than half of the publications to date have focused on understanding the magnitude of the problem [43]. The third most common physical activity research theme is correlates and determinants, which is consistent in Canada (23%) and globally (23.2%).^24^ Finally, in Canada, a larger number of articles have been published on physical activity policy than on physical activity interventions. Furthermore, the proportion of articles published on physical activity policy in Canada is 7.8%, which exactly doubles the global proportion and is higher than the 4.6% published in the PAHO (The Americas and the Caribbean) region [24]. Canada’s recognition of physical activity on its policy agenda since confederation in 1867, coupled with the country’s focus on physical activity promotion at the national level helps explain why the country has focused its efforts on policy. Additionally, as alluded to by the Toronto Charter for Physical Activity, to achieve sustainable population level changes in physical activity, both a supportive policy framework and regulatory environment are needed [44]. 

When analyzing female authorship in physical activity related research both globally and in Canada, the proportions of female first authors and female senior authors overall are similar. For instance, of the 22,656 articles that had the first and last authors listed from the systematic review by Varela et al.,^24^ 55.3% had a female first author, which is typically the position reserved for the person who contributed the most work [25, 45]. Whereas the senior author position is commonly for the principal investigator, ^45^ and 39.5% of the publications had a female in this position [25]. Of the publications in Canada, 55.0% had female first authors, and 39.4% had female senior authors respectively. Nonetheless, over the decades from the 1980s onwards, the proportion of female first authors and female senior authors continued to increase. Interestingly, of the top 10 physical activity research-producing countries (United States, Canada, Australia, Brazil, Netherlands, Spain, England, Germany, Sweden, and China), not one has greater than 50% female senior authors [25]. Addressing these gender-based inequalities in authorship is important because they can impact not only impact the ability to obtain leadership positions for females but also the opportunity for academic appointments and the capacity to secure larger grants [46, 47]. Moreover, promoting gender diversity in research contributes to increased innovation and higher quality research while also taking a crucial step towards achieving social justice [48]. 

Since the senior author position continues to be held primarily by males, more efforts focused on enhancing diversity, equity, and inclusion are needed to support the career progression of female researchers. Some strategies to consider include the development of mentorship programs that could consist of pairing senior female researchers with newer researchers to provide guidance and opportunities for career development. Previous research has shown that quality mentoring can help guide success for women involved in research in the STEM field and can also lead to both increased productivity and career satisfaction [49, 50]. Another strategy that could be explored to help close the gender gap is the creation of more family-friendly policies that include parental leave and childcare support to help ensure female researchers do not have to sacrifice their career trajectories due to caregiving responsibilities.

An interesting finding from our analysis is the apparent decline in publications from 2017 to 2019. Canada is similar to most countries in the sense that over the decades, the number of publications has increased greatly, with the most publications coming from 2010 to 2019 [24]. However, when considering the number of publications by year instead, between 2010 and 2019, the decline we see is not like other top physical activity research-producing countries. A possible explanation for this trend is a cut or lack of increase in research funding. If research labs or groups do not have the necessary funding, it may require core personnel to be laid off which could impact research capacity and quality. Likewise, insufficient funding could have caused research projects to be put on hold or even worse, could have caused research labs to be shut down altogether, leading to a decline in the number of publications. Another possible reason for this reduction is that Canada has some of the top social scientists in physical activity worldwide nowadays, and some of their publications may not be identified in the databases included in this review. Finally, a transition from surveillance to intervention studies which take longer to conduct could also help to explain this trend. However, with Canada having a long history of focusing on determinants of health and taking a population health approach opposed to running large randomized-control trials and individual level interventions, further research would be needed to draw conclusions.

Canada’s capacity for physical activity policy is considered to be high due to the existence of a standalone plan for physical activity. According to the second physical activity almanac published by the GoPA!, only 36% of countries in the PAHO region have a standalone plan [23]. Therefore, Canada continues to be an example for other nations to follow. Despite the comprehensive nature of the document, continued efforts from government and non-governmental organizations that have a stake in health promotion, such as ParticipACTION, are needed to amplify the message of physical inactivity in Canada and to help provide resources and put evidence-based recommendations into action. The strategic imperatives of The Common Vision provide a starting point and roadmap for promoting physical activity in Canada. However, increased awareness and resourcing of these strategic imperatives is crucial to building a sense of shared responsibility and encouraging the adoption of active living practices. Likewise, an additional component that could be explored when publishing future physical activity plans in Canada is the inclusion of national targets. For example, the World Health Organization’s global action plan on physical activity set out the goal of reducing global physical inactivity by 10% by 2025 and 15% by 2030 [51]. Having numeric targets in place provides concrete goals to strive for and can be used to help assess the success of physical activity promotion initiatives in Canada, similar to the Sustainable Development Goals [52]. 

Canada has several national physical activity surveillance systems, including surveys conducted by the CFLRI, the CHMS and the CCHS, which categorizes them within the 67% of the GoPA! countries to have reported having at least two national surveys that included physical activity questions [23]. Fortunately, these surveillance systems collect data from Canadians at regular time intervals. This is crucial because it can help to evaluate and assess the effectiveness of physical activity policy interventions and initiatives that are implemented in the country. However, having clear periodicity is currently not normal on the global scale, as only 30 of the 164 GoPA! members reported dates on the country cards for the first, most recent, and next survey [23]. In this regard, Canada is well equipped to both visualize physical activity trends over time and to identify disparities between different populations. In addition, Canada is one of the few countries in the world with objective assessments of physical activity at the population level, arguably having the most comprehensive physical activity surveillance in the world. Collecting objectively measured physical activity data with devices such as accelerometers is important as they are less susceptible to both recall and social desirability bias that is commonly associated with self-report measures such as surveys [53]. Consequently, when comparing physical activity levels as measured through objective and self-report measures there is often a lack of agreement [53]. Therefore, employing objective measures on a nationally representative sample of Canadians as done by the CHMS can help to more accurately depict physical activity habits of Canadians and provide a basis for future physical activity promotion efforts.

This paper has several limitations that should be acknowledged. First, in terms of determining the number of physical activity publications, the search was only conducted using PubMed, SCOPUS, and Web of Science. The use of only these databases could have led to the exclusion of relevant literature that met the inclusion criteria. Similarly, articles were included in the systematic review only if they were in English, Spanish, or Portuguese [24]. Given that Canada has two official languages, English and French, the exclusion of articles published in French could also have caused a few articles to be missed in the analysis. Additionally, the literature search only included publications up until 2019 and it is likely that more research has emerged which may offer a different perspective. For this analysis, authors were classified as male or female based on searching google, social media, government websites, and institutional websites opposed to the use of preferred pronouns. By relying on information related to sex and not gender, authors may have been misclassified. Furthermore, physical activity policy and surveillance were analyzed based on the information reported on the GoPA! Canada country card that only includes national level information.

In terms of strengths, the database from which we performed our analysis contained physical activity articles published from 1950 to 2019. The extended time frame provided a comprehensive overview of the literature and allowed us to compare publication and authorship trends in Canada and globally. Additionally, because of the work done by the GoPA! to produce country cards for 217 countries, a comparative analysis of physical activity policy and surveillance measures was made possible.

## Conclusions

Overall, Canada has continued to be a nation that places great attention on physical activity research, surveillance, and policy. While the country has made significant contributions to physical activity research globally, being a global leader, increasing population physical activity continues to be a priority. As suggested by The Common Vision, for Canada to become a country that moves more and sits less, continued collaboration and public engagement efforts are imperative. There has been an encouraging rise in females becoming first authors, although senior authorship lags in comparison. Therefore, to help achieve gender-equity in research, a collective commitment that includes all genders is crucial. In addition, strategies should be implemented to empower women to take on leadership roles and to ensure that women know that they can have enduring careers in research. Furthermore, the dissemination and support of the 24-hour movement guidelines can help to preserve health and reduce the risk for non-communicable diseases. As we work towards transforming the social climate around physical activity in Canada, smart policies and supportive environments will be necessary to foster a culture that prioritizes physical activity for all.

## Data Availability

The datasets used and/or analyzed during the current study are available from the corresponding author on reasonable request.

## Abbreviations

GoPA!: Global Observatory for Physical Activity
CHMS: Canadian Health Measures Survey
CCHS: Canadian Community Health Survey
CFLRI: Canadian Fitness and Lifestyle Research Institute
MEC: Mobile Examination Center
MVPA: Moderate–to–Vigorous Physical Activity
CAD: Canadian Dollars
NCD: Noncommunicable Disease
PAHO: The Americas and the Caribbean
